# Lessons Learned in Developing a Commercial FIV Vaccine: The Immunity Required for an Effective HIV-1 Vaccine

**DOI:** 10.3390/v10050277

**Published:** 2018-05-22

**Authors:** Bikash Sahay, Janet K. Yamamoto

**Affiliations:** Department of Infectious Diseases and Immunology, College of Veterinary Medicine, University of Florida, P.O. Box 110880, Gainesville, FL 32611-0880, USA; sahayb@ufl.edu

**Keywords:** FIV, FIV vaccine, T cell epitopes, polyfunctional T cells, cytotoxic T lymphocyte, neutralizing antibody

## Abstract

The feline immunodeficiency virus (FIV) vaccine called Fel-O-Vax^®^ FIV is the first commercial FIV vaccine released worldwide for the use in domestic cats against global FIV subtypes (A–E). This vaccine consists of inactivated dual-subtype (A plus D) FIV-infected cells, whereas its prototype vaccine consists of inactivated dual-subtype whole viruses. Both vaccines in experimental trials conferred moderate-to-substantial protection against heterologous strains from homologous and heterologous subtypes. Importantly, a recent case-control field study of Fel-O-Vax-vaccinated cats with outdoor access and ≥3 years of annual vaccine boost, resulted in a vaccine efficacy of 56% in Australia where subtype-A viruses prevail. Remarkably, this protection rate is far better than the protection rate of 31.2% observed in the best HIV-1 vaccine (RV144) trial. Current review describes the findings from the commercial and prototype vaccine trials and compares their immune correlates of protection. The studies described in this review demonstrate the overarching importance of ant-FIV T-cell immunity more than anti-FIV antibody immunity in affording protection. Thus, future efforts in developing the next generation FIV vaccine and the first effective HIV-1 vaccine should consider incorporating highly conserved protective T-cell epitopes together with the conserved protective B-cell epitopes, but without inducing adverse factors that eliminate efficacy.

## 1. Introduction

Feline immunodeficiency virus (FIV) was discovered in the fall of 1986 from a stray cat cattery in northern California [1]. Cats residing in one of the five multi-cat pens were succumbing to immunodeficiency syndromes despite being negative for feline leukemia virus (FeLV), the only immunodeficiency causing virus known at the time [2]. The new virus initially referred as feline T-lymphotropic virus; however, later renamed FIV based on its closeness with HIV [3,4,5]. FIV exhibit similar morphology and genomic organization of HIV-1 and serologically related to lentivirus than the oncogenic retrovirus [5,6,7,8,9]. FIV preferentially infect T cells with the hallmark giant cell cytopathology and encode Mg^2+^-dependent reverse transcriptase, unlike a Mn^2+^-dependent RT of FeLV [1,5]. Additionally, the infected CD4^+^ T cells die of apoptosis causing the CD4/CD8 T-cell inversion, the hallmark of all three AIDS-causing lentiviruses (HIV-1, FIV, and SIV) [4,10,11,12].

HIV-1 and FIV cause AIDS in their natural hosts, humans and domestic cats, respectively. Conversely, the simian immunodeficiency virus (SIV) live symbiotically without causing simian AIDS (SAIDS) in its natural host, the African macaques, but causes an acute manifestation of SAIDS in its unnatural hosts, the Asian macaques [13,14]. SIV was initially isolated from four Asian macaques (*Macaca mulatta* or rhesus macaques) displaying severe immunodeficiency symptoms at the New England Regional Primate Research Center [15]. The genomic sequence of SIV is close to HIV-2, whereas the SIV_CPZ_ isolated from chimpanzees is more close to HIV-1 [16]. Therefore, it has been proposed that HIV-1 originated from SIV_CPZ_ of chimpanzees, whereas HIV-2 originated from the SIV_SMM_ of African sooty mangabey [17]. Additionally, HIV-1 infected chimpanzees but did not infect other non-human primates so far tested [18]. Similar to HIV-1 and HIV-2, FIV also has African origin with close sequence similarity to wild-cat FIV_Ple_ of lions, FIV_Ppa_ of African leopards, FIV_Adu_ of cheetahs, and FIV_Ccr_ of spotted hyenas [19]. Wild-cat FIV_Pco_ subtype A and B have been found in pumas from North (subtype A), Central (subtype B) and South (subtype B) Americas, but only one wild-cat FIV_Oma_ has been identified in Pallas cats of Asia. It has been postulated that the wild-cat FIV viruses arose and evolved in African wild cats in late Pliocene (5 M–2.5 M years ago) and migrated to Asia and then to Americas during Pleistocene (126,000–12,000 years ago). A preeminent wild-cat research team claimed the African origin of the domestic cat FIV (FIV_Fca_) [19] based on the following findings: (1) The widest interspecies divergence in the *pol-RT* phylogeny exists in the wild-cat FIV_Ple_ of Africa with its divergence into six subtypes A-F, indicating a long evolution in the lion host [19,20]; (2) Africa has the most number of wild-cat FIV species [19]. Similar to HIV-1 and SIV, FIV has Africa origin and co-evolved in Africa in various feline hosts before disseminating to other global regions.

Much like the global distribution of HIV-1, FIV has disseminated in the domestic cats throughout the world [21]. The sequence analysis of FIV isolates across the world classified FIV into five subtypes (A–E) [22,23,24]. An ideal universal FIV vaccine must protect against FIV viruses from all five subtypes which is a daunting challenge as developing a universal HIV-1 vaccine for the seven HIV-1 subtypes prevailing in the world [21]. However, there are advantages of developing a lentivirus vaccine for an animal host, which is not available to human vaccines. For instance, the natural host (laboratory-bred domestic cats) can be tested directly with the experimental vaccines using live virus challenge. Another advantage is that FeLV vaccines released in U.S. in 1990–1991 were inactivated (i.e., killed) whole virus vaccines [25,26] which set a precedent of inactivated virus vaccine for future feline retroviral vaccines. In fact, the inactivated whole FeLV vaccine (Nobivac^®^ FeLV) was more effective than the recombinant canarypox virus vectored FeLV vaccine (Purevax^®^ FeLV) [27]. In addition, the long-term use of the inactivated whole FeLV vaccine demonstrated that this vaccine can be safely used in pet cats without any known incidence of FeLV infection from vaccine virus [27,28]. Since retroviruses such as FeLV may remain latently infected as provirus in the host genome, the incomplete inactivation of vaccine virus may take years before detection. In the case of FeLV vaccine, the first commercial inactivated vaccine has been available for over 20 years and are still being used due to demonstrated efficacy and safety [28]. The major concern for HIV-1 vaccine was incomplete inactivation that may lead to active infection and/or latent infection with the vaccine virus [29,30]. Consequently, no inactivated HIV-1 vaccine for prophylaxis have been tested so far in phase-I to -III human trials according to IAVA database [31]. However, inactivated HIV-1 vaccine has been tested as therapeutic vaccine in HIV-positive (HIV^+^) subjects [32].

The commercial dual-subtype FIV vaccine called Fel-O-Vax^®^ FIV was released in U.S. in 2002 and subsequently in Canada in 2003, Australia and New Zealand in 2004, and Japan in 2008 [33]. Since FIV spreads through bites and contact with the infected blood into the open lesions, intramuscular (IM) followed by intravenous (IV) transmissions are considered as the major modes of transmission more than the mucosal transmission observed in HIV [34,35,36]. Therefore, an effective commercial vaccine must protect against IM and IV transmissions. In general, more males are infected than females, and FIV infection are found more frequently in older adult cats but rarely in young kittens although cases of vertical transmission have been reported [34,37,38,39,40]. A 2011–2013 Australian serosurvey for FIV infection in client-owned cats suggested a minor decrease in FIV infection (i.e., clearly not enhanced infection) in Western Australia without major culling and termination of the FIV-positive cats after 7–9 years post release of the vaccine in Australia [37]. Since Australia has high prevalence of FIV (especially subtype A) with certain hotspots of FIV infection as great as 20% infection, Australia may be an excellent testing site for field efficacy trial of the current and the future FIV vaccines for commercial market [37].

The current review will discuss the following areas: (1) the efficacy results that led to the United States Department of Agriculture (USDA) approval of Fel-O-Vax^®^ FIV vaccine; (2) the efficacy results comparing the Fel-O-Vax^®^ FIV vaccine to the prototype dual-subtype FIV vaccine; (3) the immune mechanisms of vaccine protection observed with the commercial and prototype FIV vaccines; and (4) the recommendations to the FIV and HIV vaccine researchers that may contribute towards their effort in generating an effective second-generation FIV vaccine and the first effective HIV-1 vaccine for the global use.

## 2. Background on the Vaccine Components of the Commercial and Prototype FIV Vaccines

Both Fel-O-Vax^®^ and its prototype FIV vaccines contain inactivated two subtypes, FIV_Pet_ (subtype A) and FIV_Shi_ (subtype D) [41]. The commercial vaccine is composed of inactivated FIV_Pet_-infected whole cells (FIV_Pet_-IWC) and FIV_Shi_-IWC in FD-1 adjuvant (2 × 10^7^ cells total with about 50 µg of total viruses in the fluid). Whereas, the prototype vaccine comprises of paraformaldehyde-inactivated pelleted whole viruses (IWV) (250 µg of each virus) in the same adjuvant supplemented with either human or feline IL-12 to enhance anti-FIV T-cell immunity. Additionally, the commercial vaccine carries a higher concentration of surface envelope glycoproteins (SU Env, gp100) and/or the varying configurations of SU Env expression compared to the prototype vaccine. Consequently, the cats immunized with commercial vaccine develop higher levels of antibodies against SU Env as shown by the FIV_Pet_ immunoblot analysis of the sera of the vaccinated cats [42]. The commercial vaccine uses FIV_Pet_-infected T cell clone (FL-6) (former Fort Dodge Animal Health, Division of Wyeth) as a source of FIV_Pet_ viral components, whereas the prototype vaccine utilizes a different T cell clone (FL-4) infected with FIV_Pet_, keeping the common FIV_Shi_-infected T-cell line (FIV_Shi_/FeT-J) in both vaccines [42,43]. FIV_Pet_ virus generated from FL-6 has more intact SU Env than the virus generated from the FL-4 cell line causing the difference in antibody response between the vaccines [42]. The IL-2-independent FL-4 and FL-6 cells are clones derived from the IL-2-dependent FIV_Pet_-FeT1 cell line, whereas the FIV_Shi_/FeT-J cell line derived by infecting IL-2-independent FeT-J cells with FIV_Shi_ [44]. Since the FeT-J cell line is a derivative of the IL-2-dependent uninfected FeT1 cell line, all the cell lines in vaccine generation have a common lineage of the FeT-1 cell and do not differ in their major histocompatibility complex (MHC) allotypes. Phenotypically, FL-4 and FL-6 cells are CD3^+^CD4^−/±^CD8^+^ and lack MHC-II, whereas FeT-J and FIV_Shi_/FeT-J cells are CD3^+^CD4^−^CD8^−^ with the expression of MHC-II molecule [42]. Although the commercial vaccine has more FIV Env glycoproteins than the prototype vaccine, the prototype FIV vaccine had more protective efficacy than the commercial Fel-O-Vax^®^ FIV vaccine as described in Section 3 below.

Earlier work demonstrated equivalent protection using FIV_Pet_-infected cell vaccine (Commercial) and inactivated FIV_Pet_-IWV vaccine (Prototype) against homologous FIV_Pet_ and heterologous FIV_Dixon_ (subtype A; 11% SU and 4% TM aa sequence difference from those of FIV_Pet_) [45,46] and induced cellular immunity including T-cell immunity [46]. Furthermore, inactivated subtype-B FIV_M2_-IWC vaccine conferred complete protection of 100% (12/12) in the vaccinated cats compared to the non-vaccinated control cats (7/14) when exposed to field cats over 1.5–1.8 years [47]. These promising findings were based on experimental single-strain-IWC and -IWV vaccines. However, the commercial and prototype FIV vaccines are composed of inactivated dual-subtype A & D FIV strains to broaden the protective immunity and efficacy beyond those afforded by single-strain vaccines. Regrettably, not much is known about these vaccines in regards to the immune correlates of protection, especially in the field conditions.

## 3. Comparing the Prophylactic Efficacy of the Commercial and Prototype FIV Vaccines

Prototype vaccine containing dual-subtype IWVs demonstrated slightly better protection against heterologous subtype strains even at moderately high challenge doses than the commercial vaccine (Table 1, Studies 1–5) [21,47,48,49,50,51,52,53,54]. For example, statistically significant protection against New Zealand isolate FIV_NZ1_ (subtype F′/C) was observed with prototype vaccine (*p* = 0.0015) but not with the commercial vaccine (*p* = 0.0952) (Study 5a versus 5b). Notably, the prototype vaccine conferred significant protection when none of the vaccinated cats had detectable neutralizing antibodies (Nabs) against FIV_NZ1_ but had NAbs to FIV_Pet_ before challenge [48]. Furthermore, the NAb titers to the heterologous challenge viruses (Studies 2, 4, 5) did not correlate with the level of protection with the exception of FIV_Bang_ (Study 3). Vaccine-induced NAbs to only FIV_Pet_ and FIV_Bang_ appeared to correlate with protection observed in the vaccinated cats against the FIV_Pet_ and FIV_Bang_, respectively (Studies 1, 3), suggesting cellular immunity may be the cause of protection against other heterologous viruses, such as FIV_NZ1_. However, it is still possible that cellular immunity, such as T-cell and NK-cell activities, in combination with NAbs may work more effectively than cellular immunity alone.

Although FIV does not transmit through the mucosal route as discussed earlier, prototype IWV-vaccinated female cats conferred significant protection (83% protection, *p* = 0.0047) when challenged intravaginally with homologous FIV_Pet_, suggesting protection against mucosal challenges could be achieved when a similar strategy used in HIV-1 vaccine development where mucosa is the primary site of transmission (Table 1) [21]. The IWV-induced anti-FIV_Pet_ IgG and IgA were detected in the serum and vaginal tract but the titer of anti-FIV IgA was considerably lower than IgG [55]. As a small animal vaccine model for HIV/AIDS, this study was performed to test whether the vaccine can protect against mucosal transmission, for example sexual interaction which is the most common transmission mode for HIV-1 [36]. 

The commercial vaccine conferred no protection against systemic IM challenge [50], whereas the prototype vaccine showed some protection against systemic IV challenge with the same dose of FIV_UK8_ [48] (Table 1). Both studies showed minimal (10 NAb titer) anti-FIV_UK8_ NAb titer in a small percentage of vaccinated cats. Such low NAb titers induced by commercial vaccine did not protect any Fel-O-Vax^®^ FIV-vaccinated cats, and only one of the two IWV-vaccinated cats with a titer of 10 was among the six IWV-vaccinated protected cats [48]. Thus, no detectable NAb titer was detected in the remaining five prototype vaccinated/protected cats suggesting that vaccine-induced NAbs are not involved in vaccine protection against FIV_UK8_. Unfortunately, anti-FIV_UK8_ NAb post-challenge in Study 2b was not performed to determine any amnestic NAb levels develop shortly after FIV_UK8_ challenge, much like the observation made in a prime-boost study with canarypox virus-vectored ALVAC-FIV *gag/pro/env* prime followed by single Pet-ICV boost [56]. The difference in protection rates may be attributed to the different challenge routes used (IM versus IV) or possibly the unique feature of the FIV_UK8_. The two USDA trials used the Fel-O-Vax^®^ FIV vaccine against IM challenge with subtype-A viruses from U.S. (Table 1, Trial 10) [41,49,52] and Netherland (Trial 11) [41,49], and both trials conferred significant protection. These observations suggested that the commercial vaccine has prophylactic efficacy against subtype-A viruses in the U.S. and the Netherlands but perhaps not against subtype-A viruses in the UK. 

Since no vaccine can confer protection against excessively high challenge dose, the natural transmission dose must be determined to set appropriate challenge dose in experimental studies. Contact exposure with naturally FIV-infected cats is the ideal setting to evaluate the vaccine efficacy close to those performed in human clinical vaccine trials [33,47,51]. Such studies may take over two years for the sufficient number of infection to develop in the non-vaccinated controls, especially when the FIV prevalence is too low in the area used for natural contact exposure (Table 2) [24,33,47,51,57,58]. In one study, contact exposure of uninfected (non-vaccinated) shelter cats (9 females and 5 males) were performed in a less-restrictive free-roaming shelter facility with FIV prevalence rate of 52% (FIV incidence rate of 17%) (Table 2, Study 1) [47]. In Japan, experimental contact exposure was used in a more-restrictive closed housing system keeping uninfected and infected males together (Table 2, Study 2) [51]; additionally, in U.S. uninfected females with naturally infected males were kept together to evaluate natural transmission (Table 2, Study 3) [33]. Based on these studies, the natural transmission doses were on the average 0.48 mean cat infectious dose (CID_50_) with a range of 0.29–0.75 CID_50_ in one year of contact exposure. As expected, more uninfected cats became infected with extended contact exposure time (Table 2). Interestingly, Study 2 included an additional group of Fel-O-Vax-vaccinated laboratory cats that were all protected in the identical contact exposure to subtype-B FIV_Ao2_-infected laboratory cats (Table 1, Study 9) [51]. Such contact exposure study may be more close to the natural transmission but require 1.5 years to achieve enough infection in the control cats to determine the protection rate.

As previously discussed, hotspots of FIV prevalence (>20%) in Western Australia such as in Perth [37] may provide an ideal condition for contact exposure studies against subtype-A FIV viruses. Such a study was recently performed in Australia using client-owned cats with outdoor access from five Australian states/territories where Fel-O-Vax^®^ FIV vaccine has been available since 2004. The case-control study of 139 vaccinated cats receiving ≥3 years of annual boost and 212 non-vaccinated control cats showed protection rate of 56% upon the field exposure [54]. This protection rate was low compared to the laboratory efficacy trial performed for only 1.5 years with one annual boost (Table 1, Study 9). However, this rate observed in vaccinated cats with annual boosts was far better than the 31.2% protection rate observed in the best human HIV-1 vaccine (RV144) trial [59]. Nonetheless, to improve this efficacy of FIV vaccines beyond 56% protection, the protective immunity generated by the commercial and prototype dual-subtype FIV vaccines must be analyzed carefully towards refining and improving the commercial vaccine. To this end, the following sections attempt to determine the protective immunity and the adverse factors that can eliminate vaccine efficacy.

## 4. The Role of Vaccine-Induced Antibody (Ab) Immunity in Prophylactic Protection

The prototype IWV vaccine showed significantly more protection than the Fel-O-Vax^®^ FIV vaccine when the combined results (36/51) of the prototype vaccine was compared to the combined results (11/24) of the Fel-O-Vax^®^ FIV vaccine (*p* = 0.0453) (Table 1). The percentage protection of each vaccine did not correlate with the level of NAbs produced by the vaccine to the challenge virus with exception to homologous FIV_Pet_ and heterologous FIV_Bang_. The commercial vaccine produced FIV NAbs more consistently in more vaccinated cats and generally at higher titers than the prototype vaccine (Table 1). The studies comparing the NAb titers between these vaccines demonstrated quite complex findings [48]. The commercial vaccine induced two-fold higher average NAb titers to homologous FIV_Pet_ but 23% lower percent responders (percent of total animals showed titer ≥10 upon vaccination) with NAbs than the prototype vaccine (Table 1 and Table 3). (Table 3 includes the summarized results of Table 1 and three additional NAb results to FIV_Dix_ (subtype A), FIV_MD_ subtype B), and vaccine strain FIV_Shi_ (subtype D). Both vaccines induced more NAb titers to FIV_Pet_ than those to FIV_Shi_ (Table 3). Likewise, both vaccines had similar percent NAb responders to FIV_Shi_ but the average NAb titer to FIV_Shi_ of the prototype vaccine was higher than that of the commercial vaccine. This trend differed from those to FIV_Pet_ (Table 3).

In another study, the dual-subtype FIV_Pet_-ICV (FL-4) plus FIV_Shi_-ICV (FIV_Shi_-FeT-1) vaccine conferred 100% protection against FIV_Shi_ when neither single-virus FIV_Pet_-ICV nor FIV_Shi_-ICV was able to achieve such protection [60]. Notably, only 2/4 dual-subtype vaccinated cats had NAb titer to FIV_Pet_ before FIV_Shi_ challenge, whereas none (0/4) had NAbs to FIV_Shi_. These observations suggested that the dual-subtype vaccine, but not single-virus vaccines, had a synergistic effect on efficacy and perhaps on the types of immunity produced and that the vaccine-induced NAb to FIV_Shi_ did not correlate with protection. The latter assumption is made with a cautionary note since the authors of this article did not evaluate the NAb titers to FIV_Shi_ after challenge. Since their assay did not detect NAb levels of below 10, the possibility exists for extremely low titers of anti-FIV_Shi_ NAbs present before challenge may be detected post-challenge as an amnestic response which can potentially clear the FIV_Shi_ infection. Such anamnestic response has been previously observed in ALVAC study [56].

Most importantly, both vaccines produced low average NAb titers (<5–20) to heterologous strains, and the commercial vaccine produced slightly more percent NAb responders to challenge virus vaccine than the prototype vaccine (Table 1). The NAb titers to subtype-A FIV_UK8_ were ≤10 in all vaccinated cats before the challenge even though high Nab titers to FIV_Pet_ which also belong to subtype A. Both vaccines developed low NAb titers to FIV_UK8_ in a low number of Nab responders. Furthermore, these vaccines conferred either no protection (commercial vaccine) or marginal protection (prototype vaccine). In contrast, both vaccines afforded significant protection to heterologous subtype-B FIV_FC1_ (Table 1, Study 4) even with the similarly low level of NAbs to FIV_FC1_ in a small percentage of vaccinated cats. 

Passive transfer studies using the pooled sera and purified antibodies from Fel-O-Vax-vaccinated cats were performed on naïve specific-pathogen-free (SPF) cats against challenge infection with either FIV_Pet_ or FIV_FC1_ [48]. The passive transfer of vaccine antibodies with high titers of anti-FIV_Pet_ NAbs conferred significant protection against homologous FIV_Pet_ but conferred no protection against heterologous FIV_FC1_ (Table 4). These findings suggested that protection against vaccine virus FIV_Pet_ was mainly mediated by antibody immunity including NAbs. In contrast, the protection against distinctly heterologous subtype-B FIV_FC1_ was not mediated by the vaccine-induced antibody immunity. The latter finding is noteworthy since prototype vaccine afforded 100% protection against FIV_FC1_ (Table 1, Study 4b). Since prototype vaccine-induced FIV-specific CTL and polyfunctional T-cell activities [61,62], the likelihood of vaccine-induced T-cell immunity playing a major role in protection against heterologous subtype viruses was a strong possibility.

## 5. The Role of Vaccine-Induced T-Cell Immunity in Prophylactic Protection

The first prime-boost FIV vaccine study held in 1996 provided the first indication of the role of T-cell immunity in vaccine protection [56]. The 2X ALVAC (canary poxvirus)-vectored subtype-A FIV_Ville Frache_ *gag/pro/env* prime followed by a single ICV-FIV_Pet_ boost conferred slightly more protection against FIV_Pet_ (100% vs. 67%) than those boosted with ALVAC-FIV *gag/pro/env*; however, only three animals per group were tested to make such conclusion (Table 5 adapted from [56]). Before FIV_Pet_ challenge (50 CID_50_), all cats in ICV-FIV_Pet_ boost group and two cats in ALVAC-FIV boost group developed FIV antigen-specific cytotoxic T lymphocyte (CTL) and/or T-helper activity but did not develop any detectable anti-FIV_Pet_ NAbs. The anti-p24 Ab titers were very high in the ICV boost group and in the control group that received empty ALVAC vector prime with ICV boost, but none detected in the ALVAC-FIV boost group. At 8 months post-first challenge, the protected/vaccinated cats and one non-vaccinated control cat without additional boost were challenged with a high dose (75 CID_50_) of recombinant subtype-A*_gag-pol_*/B*_env_* FIV_Bang_. All cats in the ICV-FIV_Pet_ group was protected, but no protection was observed in the ALVAC-FIV *gag/pro/env* group and the single control cat. Before the second challenge, only one protected cat had low levels of FIV_Pet_ and FIV_Bang_ titers, whereas the others had no NAb titer to either virus. All protected cats developed no NAbs to FIV_Bang_ post-FIV_Bang_ challenge, and therefore, such protection was not mediated by amnestic anti-FIV_Bang_ NAbs. The authors concluded that vaccine-induced NAbs and T-cell immunity are important in prophylactic protection against FIV_Pet_ which had Env sequence closely related to the vaccine strain (3% and 1% differences in Env and Gag aa sequences, respectively) [56]. The low levels of anti-FIV_Pet_ NAbs which developed or persisted after FIV_Bang_ challenge, suggested a possibility of the synergistic role of non-NAbs and anti-FIV T-cell immunity in protection [55]. This assumption was strengthened with the recent phase-III HIV vaccine trial (RV144 trial) that showed 31.2% protection in the overall risk group [59]. This protection correlated or associated with high vaccine-induced antibody-dependent cellular cytotoxicity (ADCC) Abs and anti-V2(Env) CD4^+^ T-cell immunity including polyfunctional CD4^+^ T cells and CD4^+^ CTLs [59,63,64]. Moreover, in a failed phase-III AIDSVAX B/E (VAX004) vaccine trial, HIV-specific ADCC-Abs, but not Nabs, were associated with lower infection risk [65,66]. These HIV vaccine trials suggested that ADCC Abs can play a role in vaccine protection when only low levels of NAbs are detected [66,67].

The ALVAC-FIV prime-boost study (Table 5) and the commercial and prototype vaccine trials (Table 1) did not test for vaccine-induced ADCC-Abs. Hence, ADCC-Abs together with anti-FIV T-cell immunity and even low titers of NAbs may play a role in vaccine protection against heterologous FIV challenge. The initial T-cell studies consisted of adoptive transfer (AT) of washed blood cells containing 1–2 × 10^7^ peripheral blood mononuclear cells (PBMC) from a subtype-A FIV_Pet_-IWV vaccinated parent (AT donor) to MHC half-matched offspring (AT recipient) to prevent rejection of the immune cells by the AT recipients (Table 6, Studies 1–3) [68]. Only MHC half-matched or identical matched recipients of blood immune cells showed partial-to-complete AT protection against homologous FIV_Pet_ challenge. Partial protection was defined as no development of FIV-p24 antibodies (Abs) and a major delay in virus isolation from PBMC throughout 24–32 weeks post-challenge (wpc). None of the AT recipients had p24 Abs at 1 wpc, indicating that the p24 Abs were washed off and the transfer of p24-specific B and the plasma cells were minimal during the adoptive transfer. When the results from Studies 1–3 were combined and compared to the protection rate of the combined control group, significant protection rate was achieved in 10/13 partial-to-complete protection (*p* < 0.0001) and 6/13 complete protection (*p* = 0.0013). The combined control group consisted of nonvaccinated/half-matched, nonvaccinated/unrelated and vaccinated/unrelated recipients, and nonvaccinated/non-AT cats. These studies indicated that the AT protection was MHC restricted and that the transferred FIV-specific T cells from donor prevented FIV infection in the AT recipient when the MHC was at least partially matched between the donor and recipient.

Above studies, however, did not demonstrate whether the transferred vaccine-induced memory B cells were working in synergy with the transferred anti-FIV T cells. In recent studies, purified T and B cells from vaccinated AT donors were adoptively transferred to partial-to-compete MHC matched recipients (Table 6, Studies 4–7) [62]. In Study 4 that was based on MHC matching by mixed leukocyte reaction (MLR) (Table 6, Study 4), 3/4 (75%) AT recipients of MLR-matched immune T cells were protected, whereas none (0/4) of the recipients receiving either MLR-matched immune B cells or unrelated non-immune PBMC were protected. Based on MHC-I, MHC-II, and MLR matching (Study 5), the recipients of the completely-matched immune T cells were all (4/4) protected against homologous FIV_Pet_ challenges, whereas partial protection was observed (2/3) in the recipients each of either partial-to-completely-matched immune CD8^+^ T cells or CD4^+^ T cells. In this study, one recipient of partially MHC-matched immune CD8^+^ T cells, and another recipient of completely MHC-matched immune CD4^+^ T cells were infected. Thus, complete AT protection was consistently observed when both immune CD4^+^ and CD8^+^ T cells were transferred together as a whole T-cell population, and when the donor and recipient were completely MHC matched. This study further suggested that the prophylactic protection of prototype vaccine is mediated by vaccine-induced T-cell immunity which appears to require both CD4^+^ and CD8^+^ T-cell immunity to exert complete protection against homologous FIV_Pet_ challenge. In Study 6, all recipients (5/5) of completely MHC-matched immune T cells were protected again, resulting in a combined protection rate of 100% (9/9) in the AT recipients of completely MHC-matched T cells from Studies 5 and 6 (*p* < 0.0001) (Table 6). Current results together with those from humoral and passive immunity studies support the concept that the vaccine should induce both B-cell and T-cell immunity in synergy to confer complete protection against FIV strains closely related in the Env sequence to subtype-A and -D vaccine strains.

## 6. Determining the Importance of T-Cell Immunity Produced by the Prototype Vaccine

In the humoral immunity studies described earlier, vaccine-induced minimal-to-nil titers of NAbs were detected against heterologous subtype (B and F′/C; Table 1) strains. These findings suggested that immunity other than B-cell immunity were important in mounting the protective response observed against viruses of heterologous subtypes. Passive transfer (PT) of purified antibodies did not confer protection in PT recipients when challenged with heterologous subtype-B FIV_FC1_, but significant protection was observed when challenged with homologous FIV_Pet_. This observation demonstrated that FIV_FC1_ is resistant to Fel-O-Vax-induced Abs and any other Ab-mediated antiviral activity (e.g., ADCC-Abs). This finding also suggested that the immunity other than Ab immunity was the real cause for the protection against heterologous challenges (e.g., FIV_FC1_). This assumption was confirmed when purified T cells from prototype IWV-vaccinated cats showed significant AT protection in (6/9) recipients of completely MHC-matched immune T cells but no protection in the control recipients of non-immune/MHC-unmatched T cells, PBS, or without AT (0/8) (*p* = 0.009). The authors concluded that the AT protection was mediated by vaccine-induced T cells based on the following three observations: (1) the AT preparation of the purified T cells contained minimal-to-nil contaminating B cells; (2) no vaccine-induced antibodies were detected before or at 3 wpc, the initial time when only one control cat developed FIV immunoblot Abs; and (3) AT protection required complete MHC matching, demonstrating MHC-restricted T-cell immunity instead of MHC-unrestricted Ab immunity [62]. Hence, vaccine-induced T-cell immunity is vital for the prophylactic protection afforded by the prototype IWV vaccine as well as perhaps by the Fel-O-Vax^®^ FIV vaccine. The latter is most likely the case since Fel-O-Vax-induced Abs had no PT efficacy against FIV_FC1_, nevertheless, the vaccine attained significant (71.8–75.0%) prophylactic protection against FIV_FC1_ (Table 3).

The FIV-specific T-cell immunity generated by the prototype vaccine consisted of FIV-specific polyfunctional CD4^+^ T-cell activities (perforin, granzyme A, granzyme H, TNFα, IFNγ, IL-2, IL-4, IL-10), polyfunctional CD8^+^ T-cell activities (perforin, granzyme A, granzyme B, granzyme H, IFNγ, IL-2), CD4^+^ CTL activities (perforin, granzymes A and H), CD8^+^ CTL activities (perforin, granzymes A, B, H), and FIV-specific CD4^+^ and CD8^+^ T-cell proliferation responses [61,62]. A note of caution must be placed on IFNγ and possibly on TNFα. Overproduction of these two cytokines may be concerning due to their pro-inflammatory nature that could enhance HIV-1 replication in the host (described in review [69]) as well in primary macrophage, CD4^+^ cells, and PBMC [70]. In the failed phase-IIb HIV vaccine (STEP) trial, the non-specific IFNγ secretion was associated with increased HIV infection risk [71]. The addition of feline IFNγ together with FIV enhanced the FIV replication in primary PBMC cultures [72]. Likewise, treatment with human IFNγ enhanced HIV-1 replication in human PBMC [73]. Furthermore, in an adjuvant study using the same dual-subtype IWV immunogen as the commercial and prototype vaccines, IWV-vaccinated cats with adjuvants other than FD-1 adjuvant were less protective when their PBMC had higher levels of FIV-specific IFNγ production than those from the prototype IWV-vaccinated cats [74]. However, a moderate production of IFNγ and TNFα together with IL-2, perforin, and granzymes may be beneficial as long as inflammatory cytokines are not overproduced [69].

## 7. A Novel Approach to Confirm the Value of T-Cell Immunity in Conferring Protection

The FIV peptides that induce potent CD8^+^ CTL, CD4^+^ CTL, and polyfunctional CD4^+^ and CD8^+^ T-cell activities, along with strong FIV-specific CD4^+^ and CD8^+^ T-cell proliferation responses should be included as vaccine immunogens. The FIV epitopes that induce these protective activities should be short linear peptides for T-cell recognition. T cells only recognize the peptides presented on the MHC-I molecules of infected cells or MHC-II molecules of the antigen presenting cells (APC) (e.g., macrophages, dendritic cells, lesser extent by B cells) [75]. More importantly, such vaccine peptide epitopes should be conserved among FIV subtypes and induce protective T-cell immunity without generating escape variants. Therefore, the protective conserved FIV epitopes for an effective vaccine must be essential to the viral survival since any substantial mutation(s) should affect the fitness of the virus [76,77,78]. 

The conserved epitopic (8–15mer aa) sequences among the FIV viruses within the same subtype and even among the different subtypes provide numerous potential intra-subtype and inter-subtype conserved epitopes but do not identify epitopes which resist mutation or induce protective T-cell responses. One approach in focusing potential T-cell epitopes on FIV proteins is to identify those epitopes that are conserved among two or three AIDS lentiviruses (HIV-1, FIV, SIV) and that possess the ability to induce anti-FIV T-cell activity(s) [79,80]. Furthermore, such conserved epitopes are most likely to have withstood the evolutionary pressure in their respective host but have been retained as part of the virus since any mutation(s) most likely affected the survival of the virus. The first step in pursuing this approach of T-cell epitope selection is to identify the most conserved FIV/HIV-1 proteins. 

The most conserved proteins on the FIV was determined by comparing the sequences of HIV-1 and FIV and identified to be Pol-RT (polymerase-RT, 72.0%) followed by Pol-IN (Pol-integrase, 64.7%) and Gag-CA/p24 (Gag-capsid or p24, 63.1%) [79]. This evolutionary approach in the assessment of conserved AIDS virus epitopes was validated by the findings of others who compared genes and amino acid (aa) sequences of domestic-cat FIV to wild-cat FIVs. The genetic divergence of lion FIV_PLe_ subtype E compared to Pallis-cat FIV_Oma_, puma FIV_Pco_, and domestic-cat FIV determined that FIV *gag*/Gag and *pol*/Pol genes and aa are more conserved than FIV *vif*/Vif, *orfA*/OrfA, and *env*/Env, suggesting that host selection or immune pressure may more readily mutate *vif*, *orfA*, and *env* genes without affecting viral replication [19]. Similar analyses have been performed with different FIV isolates from domestic cats, which also shows that Gag sequences (Pol sequence not tested) are more conserved than Env sequences [58]. Similarly, protective conserved HIV-1 epitopes were identified predominantly on Gag and Pol when compared to those on Env, Nef and accessory proteins [81,82,83,84]. Hence, major changes by mutations in the *gag* and *pol* genes may affect the survival of the virus. Since Gag-p24 and Pol-RT antigens are not involved in inducing anti-FIV/HIV neutralizing antibodies (NAbs), immunoprotective epitopes on Gag and Pol proteins may in place induce FIV/HIV-specific T-cell immunity such as anti-FIV/HIV CD8^+^ cytotoxic T lymphocytes (CTLs), CD4^+^ CTLs, and polyfunctional T cells [85]. However, simply vaccinating with recombinant Gag and/or Pol proteins or with a vector containing FIV *gag/pol/env* genes have been unsuccessful in conferring protection against FIV challenge [41,49,86]. Such results suggest that selecting protective T-cell epitopes from the Gag-p24 and Pol-RT may be a better strategy since expression of whole viral proteins do not appear to confer protection. The need for such a selection also infers that viral proteins are composed of epitopes that enhance viral infection as well as epitopes that readily mutate to escape vaccine immunity [79,85,87,88]. The former case has been demonstrated in a preliminary in vitro study which demonstrated the presence of FIV enhancing epitope nearby a protective T-cell epitope on FIV p24 [89]. 

The T-cell functional parameters derived from those observed in the protected IWV-vaccinated cats have been used to identify the conserved T-cell epitopes on FIV p24 and RT that stimulate anti-FIV/HIV T-cell activities in the PBMC of both HIV^+^ human subjects and IWV-vaccinated cats [87,88]. Two FIV p24 peptides and two FIV RT peptides were selected by this method [89]. These peptides were combined in a multiple antigenic peptide (MAP) formulation with a lysine backbone linked to four identical peptides. The combined MAP vaccine consisted of four MAPs (100–120 µg per MAP; a total of 400–480 combined MAPs) in FD-1 adjuvant [89]. The MAP vaccine conferred protection in 15/19 vaccinated cats against subtype-B FIV_FC1_ challenge, whereas 10/16 control cats were infected (% Preventable Fraction of 66.3%, *p* = 0.0181). Since no Env peptide was used in this study, T cells are most likely mediating the vaccine protection against FIV. In support of this assessment, the protected/vaccinated cats developed strong FIV-specific T-cell immunity to the p24 and RT peptide epitopes in the MAP vaccine before challenge [89]. This preliminary study demonstrates that the T-cell immunity induced by the prototype vaccine is essential in the successful prophylaxis and more likely this may be the case for Fel-O-Vax^®^ FIV vaccine.

## 8. Summary of Lessons Learned in Developing a Commercial and Prototype FIV Vaccines

The major lesson learned from understanding the immune mechanisms of protection provided by the prototype vaccine is the need for the vaccine to produce high levels of multiple anti-FIV T-cell responses to confer protection against all subtypes of FIV. Much like HIV-1 vaccine research, current efforts on developing a T cell-based FIV vaccine has been insufficient since the major focus of vaccine research has been to develop a B cell-based or an Ab-based FIV vaccine [31,41,49,80,86]. Furthermore, the failed vaccine trials to develop a consistent and high levels of anti-FIV T-cell immunity used vector vaccines with FIV *gag/pol* containing constructs, inactivated whole-virus (FIV_UK8_, FIV_UT113_) vaccines, and proviral DNA RT and IN which should have generated FIV Gag- and Pol-specific T-cell responses [41,49,56]. Therefore, one major challenge is to weed out non-protective, replication enhancing epitopes and to select for evolutionary conserved (non-mutable), protective T-cell epitopes on FIV Gag and Pol. Another challenge is to identify an approach which combines T-cell epitopes of FIV Gag and Pol with broad NAb (bNAb)- and ADCC-Ab-inducing B-cell epitopes on FIV Env, without inducing blocking Abs to the NAb and ADCC epitopes and without stimulating excessive CD4^+^ T-cell activation and cytokines which enhance viral replication [85,89]. Overall, the task of developing FIV and HIV vaccines with selected protective B- and T-cell epitopes will require major innovations at the selection of the epitopes, developing a strategy in combining of the B-cell and T-cell epitopes, and choosing the appropriate vaccine delivery platform targeting the APCs. Lessons learned from the commercial and the prototype FIV vaccines indicate that most of their vaccine protection was mediated by anti-FIV T cell immunity more than anti-FIV Ab immunity (NAbs, ADCC-Abs). However, inducing both T- and B-cell immunity against all subtypes of FIV will be an ideal scenario. Thus, it is anticipated that a highly effective HIV vaccine will need to produce potent anti-HIV T-cell immunity in addition to protective anti-HIV Abs (NAbs, bNAbs, ADCC-Abs), but without concomitant production of blocking antibodies and HIV-enhancing cytokines.

## Figures and Tables

**Table 1:** Comparing the FIV neutralizing antibody (NAb) titer and prophylactic efficacy of the commercial and the prototype FIV vaccines.

Study ^a^	Vaccine ^a^	Challenge Strain ^b^		Ave NAb Titer ^c^ (% Responder)	Protection Rate of Vaccinee (%)	Protection Rate of Control (%)	% Preventable Fraction (*p*-Value) ^d^	References
		Subtype	Route (CID_50_)					
Short-Duration Studies (3 Vaccinations)								
1A	Fel-O-Vax	FIV-Pet (A)	IV (50)	216 (63%)	5/5 (100%)	0/8 (0%)	100% **(*0.0008*)**	[48,49]
1B	IWV	FIV-Pet (A)	IV (25)	118 (83%)	6/6 (100%)	0/6 (0%)	100% **(*0.0022*)**	[48,49]
2A	Fel-O-Vax	FIV-UK8 (A)	IM (10)	10 (30%) ^e^	0/6 (0%)	0/5 (0%)	0.0% (*1.0000*)	[48,50] ^e^
2B	IWV	FIV-UK8 (A)	IV (10)	10 (13%)	6/15 (40%)	0/15 (0%)	40.0% **(*0.0169*)**	[48]
3A	Fel-O-Vax	FIV-Bang (A/B)	IV (10–25)	22 (70%)	1/4 (25%)	0/4 (0%)	25.0% (*1.0000*)	[48]
3B	IWV	FIV-Bang (A/B)	IV (25)	73 (61%)	10/14 (71%)	0/10 (0%)	71.4% **(*0.0006*)**	[48]
4A	Fel-O-Vax	FIV-FC1 (B)	IV (15)	10 (30%)	3/4 (75%)	0/10 (0%)	75.0% **(*0.0110*)**	[48]
4B	IWV	FIV-FC1 (B)	IV (15)	10 (13%)	8/8 (100%)	0/10 (0%)	100% **(*<0.0001*)**	[48]
5A	Fel-O-Vax	FIV-NZ1 (F′/C)	IV (50)	10 (20%)	2/5 (40%)	0/10 (0%)	40.0% (*0.0952*)	[48]
5B	IWV	FIV-NZ1 (F′/C)	IV (50)	<5 (0%)	6/8 (75%)	0/10 (0%)	75.0% **(*0.0015*)**	[48]
	*Combined Fel-O-Vax FIV Vaccine:* ^f^				*1/24 (46%)* ^f^	*0/37 (0%)* ^f^	*45.8%* **(*<0.0001*)**	
	*Combined Prototype IWV Vaccine:* ^f^				*36/51 (71%)* ^f^	*0/51 (0%)* ^f^	*70.6%* **(*<0.0001*)**	
6	IWV	FIV-Pet (A)	Vag (25)	NA	5/6 (83%)	0/7 (0%)	83.3% **(*0.0047*)**	[21]
Long-Duration Studies (3 Vaccinations plus 1-yr Boost)								
7	Fel-O-Vax	FIV-Bang (A/B)	IV (25)	50	2/4 (50%)	0/3 (0%)	50.0% (*0.4286*)	[48]
8	Fel-O-Vax	FIV-FC1 (B)	IV (25)	<5	6/13 (46%)	0/5 (0%)	46.2% (*0.1141*)	[48]
1.5-Year Contact Study (3 Vaccinations plus 1-yr Boost)								
9	Fel-O-Vax	FIV-Ao2 (B)	CE (0.87) ^g^	NA	12/12 (100%)	9/16 (62%)	100% **(*0.0103*)**	[51]
1-Year Challenge Studies without Boost (3 Vaccinations)								
10-USDA	Fel-O-Vax	U.S. (A)	IM (1.58) ^g^	NA	39/52 (75%)	11/53 (26%)	68.5% **(*<0.0001*)**	[41,49,52]
11-USDA	Fel-O-Vax	Dutch (A)	IM (1.73) ^g^	NA	21/24 (87%)	2/15 (13%)	85.6% **(*<0.0001*)**	[41,49]
12	Fel-O-Vax	FIV-FC1 (B)	IV (>2.0)	NA	10/14 (71%)	0/5 (0%)	71.8% **(*0.0108*)**	[53]
Australian Field Study on Client-Owned Cats with Annual Boost for >3 yr								
13	Fel-O-Vax	Australia (A)	CE (0.24) ^g^	NA	84/89 (94%)	187/212 (88%)	56.0% ^h^ (*0.14000*)	[54]

^a^ Studies 10 and 11 for USDA submission; commercial dual-subtype FIV vaccine (Fel-O-Vax); prototype dual-subtype FIV vaccine IWV). ^b^ U.S. isolates (California FIV_Pet_, Boston recombinant subtype-A*_gag-pol_*/B*_env_* FIV_Bang_, Florida FIV_FC1_); Glasgow FIV_UK8_; New Zealand recombinant subtype-F’*_gag-pol_*/C*_env_* FIV_NZ1_ (F′/C); Japan FIV_Ao2_; U.S. and Dutch isolates in Studies 10–11 (proprietary information). ^c^ Average NAb titer to challenge virus (Ave NAb titer) from reference [48]. ^d^ % Preventable Fraction = (% infected control − % infected vaccinate)/(% infected control); two-tailed Fisher Exact Test for *p*-value (italics) with *p* < 0.05 as significant (bolded italics). ^e^ Study 2A from reference [50] provides no NAb information and the average NAb to FIV_UK8_ is from reference [48]. ^f^ Combined Fel-O-Vax vaccine results from Studies 1A, 2A, 3A, 4A, and 5A; combined prototype IWV vaccine results from Studies 1B, 2B, 3B, 4B, and 5B. ^g^ Transmission dose of the contact exposure (CE) Studied 9 & 13 and the Challenge Studies 10 and 11 are derived from the results of the control group. ^h^ Protective efficacy based on publication [54].

**Table 2:** Natural transmission dose based on contact exposure studies before Fel-O-Vax^®^ FIV release in the respective countries ^a.^

Trial/Study ^b^ (Reference)	Type of Contact Exposure	Years of Contact Exposure	Transmission Rate (%)	Transmission Dose (CID_50_) ^c^	FIV Donor Subtype	Subtype Prevalent in Country ^d^ (References)
Italy [47] 2000	Free roaming (field FIV+ cats)	1 1.5–1.8	2/14 (14%) 5/14 (36%)	0.29 0.72	Subtype B	Subtype B [24,57]
Japan [51] 2005	Closed housing ^e^ (FIV_Ao2_-infected males)	1 1.5	3/8 (37%) 4/8 (50%)	0.75 1.00	Subtype B (FIV_Ao2_) ^e^	Subtypes B, A, D [58]
U.S. [33] 1988	Closed housing ^f^ (Mixed-sex field FIV^+^ cats from CA & HI)	1 1.75 2.5	1/3 (33%) 2/3 (67%) 3/3 (100%)	0.67 1.33 2.00	Subtype A Subtype B	Subtypes B, A [24]
Combined Studies	-	1 1.5–1.8 2.5	6/25 (24%) 11/25 (44%) 3/3 (100%)	0.48 0.88 2.00	Subtype A Subtype B	

^a^ Table adapted from reference [33]. ^b^ Contact exposure trials in three countries shown with either the publication date of the trial (Italy, Japan) or the start date of the study (U.S.) were performed before Fel-O-Vax^®^ FIV release in the respective countries. ^c^ Mean (50%) cat infectious dose (CID_50_). ^d^ Subtypes in the order of prevalence in the country. ^e^ FIV_Ao2_-infected male cats were housed with FIV-naïve male cats.

**Table 3:** Average NAb to vaccine viruses and heterologous subtype-A FIV viruses ^a,b^.

FIV Strain (Subtype) ^c^	Location of FIV Isolation	Ave Fel-O-Vax NAb Titer (% Responder) ^d^	Ave IWV NAb Titer (% Responder) ^d^	Combined % Preventable Fraction	
				Fel-O-Vax ^d^	IWV ^d^
**FIV-Pet (A)**	Petaluma CA, U.S.	**216** (63%)	**118** (83%)	**100%**	**100%**
**FIV-Shi (D)**	Shizuoka, Japan	**60** (74%)	**96** (78%)	**100%** ^b^	--
FIV-Dix (A)	Dixon CA, U.S.	--	**80** (70%) ^b^	--	--
FIV-U.S.(A)	U.S.	--	--	**68.5%**	--
FIV-UK8 (A)	Glasgow, UK	10 (30%)	10 (13%)	0%	40%
FIV-Dutch (A)	Netherland	--	--	**85.6%**	--
FIV-Bang (A/B)	Boston, MA, U.S.	22 (70%)	73 (61%)	25.0%	**71.4%**
FIV-FC1 (B)	Florida, U.S.	10 (30%)	10 (13%)	**71.8–75.0%**	**100%**
FIV-MD (B)	Maryland, U.S.	20 (62%) ^b^	10 (36%) ^b^	--	--
FIV-Ao2 (B)	Aomori, Japan	--	--	100% ^b^	--
FIV-NZ1 (F′/C)	New Zealand	10 (20%)	<5 (0%)	40.0%	**75.0%**

^a^Table 3 is the summary of Table 1 with emphasis on NAb titers generated by Fel-O-Vax^®^ FIV vaccine and prototype IWV vaccine. ^b^ Additional data provided are on the average NAb titers to FIV_Dix_ [55] and to FIV_MD_ [49], and on the dual-subtype FIV_Pet_-ICV/FIV_Shi_-ICV vaccine against FIV_Shi_ challenge [60]. ^c^ Vaccine strains (bolded). ^d^ Substantial NAb titers (bolded) or significant percent preventative fraction (bolded) when *p* < 0.05; Not available (--).

**Table 4:** Fel-O-Vax-induced Ab immunity in passive-transfer (PT) protection against homologous FIV_Pet_ and heterologous subtype-B FIV_FC1_^a.^

PT Study	PT of Serum or Abs	Anti-Pet NAb Titer (Range) ^b^	Anti-FC1 Nab Titer (Range) ^b^	Challenge (25 CID_50_)	Protection Rate (*p*-Value) ^c,d^	Combined Protection Rate (*p*-Value) ^c,d^
1A1	Fel-O-Vax-induced pooled serum Abs	513 (50–1000)	<5	FIV-Pet	4/4 **(*0.0286*)**	11/12 **(*<0.0001*)**
1A2	Fel-O-Vax-induced purified Abs	639 (10–1000)	50 in only 1 cat ^e^	FIV-Pet	7/8 **(*0.0014*)**	-
1B1	Pooled control cat serum or saline	<5	<5	FIV-Pet	0/4	0/11
1B2	Purified non-vaccine Abs or saline	<5	<5	FIV-Pet	0/7	-
2A	Fel-O-Vax-induced purified Abs	630 (50–1000)	30 in only 2 cats ^e^	FIV-FC1	0/5 (NS)	0/5 (NS)
2B	Purified non-vaccine Abs or saline	<5	<5	FIV-FC1	0/5	0/5

^a^Table 4 adapted from reference [48]. ^b^ Average neutralizing antibody (NAb) titer and range to either FIV_Pet_ (Pet) or FIV_FC1_ (FC1) present in PT cats at 6 days post-PT or 1 week post-challenge. ^c^ Comparing Groups A *versus* B in a two-tailed Fisher Exact Test for *p*-value (italics) with *p* < 0.05 as significant (bolded italics); not significant (NS). ^d^ Statistics based on 1A1 vs. 1B1 (*p* = 0.0286), 1A2 vs. 1B2 (*p* = 0.0014), or A vs. B (*p* < 0.0001) or (NS). ^e^ Only one cat in Group 1A2 and two cats in Group 2A had anti-FIV_FC1_ NAb titer of 50 and 30, respectively.

**Table 5:** Prime-boost Pilot Study with ALVAC-vectored subtype-A FIV_VilleFranche_ *gag/pro/env* prime and FIV_Pet_-ICV boost ^a^.

			Post-Vaccination or Pre-FIV_Bang_ Immune Status ^b,c^				Post-Challenge Immune Status ^c,d^			
Group (# of Cats)	Vaccine (2×)	Boost (1×)	WB Titer (Range)	Pet NAb Titer ^e^ [FC1]	% CTL ^e^ (Range)	T-helper (SI) ^e^	WB titer ^e^ (Range)	Pet NAb Titer [FC1] ^e^	T-Helper (SI) ^e^	Protection Rate
1st Challenge FIV-Pet (50 CID_50_)										
1 (3)	ALVAC-FIV	ICV	+ (5–6)	<5 [N]	27/55/8 (8–55)	N/N/2.0	+ (4–5)	5–20	N/N/1.3	3/3
2 (3)	ALVAC-FIV	ALVAC-FIV	− (<2)	<5 [N]	7/18/37 (7–37)	N/2.5/1.5	<2/<2/5–6	>100/N/<5	1.8/N/N	2/3
3A (3)	ALVAC	ICV	+ (3–5)	<5 [N]	5/N/N (NA)	1.1/1.3/N	+ (5–6)	≥100	1.2/N/N	0/3
2nd Challenge FIV-Bang (75 CID_50_; 8 mo post-1st Challenge)										
1 (3)	Above ^f^	Above ^f^	+ (4–5)	<5/<5/5–20 [<5]	N	N	+ (3–4)	5–20 [<5]	N	3/3
2 (2)	Above ^f^	Above ^f^	− (<2)	<5 [<5]	N	N	+ (4–5)	N [<5/>100]	N	0/2
3B (1)	None ^f^	None ^f^	− (<2)	N [<5]	N	N	− (4)	N [<5]	N	0/1

^a^Table 5 adapted and summarized from [56]. ^b^ Immune status of post-vaccination before 1st challenge (FIV_Pet_) at 50 CID_50_ on the top three rows and immune status of pre-2nd challenge (FIV_Bang_) at 75 CID_50_ on the bottom three rows. ^c^ Western blot (WB) titer based on positive (+) or negative (−) with dilution titer range; anti-FIV_Pet_ neutralizing antibody (Pet NAb) titer; anti-FIV_FC1_ NAb [FC1] titer; percent cytotoxic T lymphocyte activity (% CTL); T-helper cells (T-helper) based on FIV-specific proliferation shown as stimulation index (SI); not tested (N). ^d^ Immune status of post-1st challenge on the top and viral-specific antibody status of post-2nd challenge on the bottom. ^e^ Individual cat result shown as (cat-1 result/cat-2 result/cat-3 result) and all are shown in same order for the group. ^f^ All protected cats in Groups 1 and 2 without a boost received the 2nd challenge at 8 months post-1st challenge along with one age-matched SPF cat (Group 3B).

**Table 6:** Adoptive transfer (AT) Studies to test the role of immune T cells from prototype IWV-vaccinated cats ^a.^

Study/Group (Reference)	AT Donor	Number of AT Recipient ^b^	Type of MHC Matching ^c^	AT Cell Type ^d^	Challenge FIV (CID_50_) ^e^	P-C Protect Rate [A vs. B, *p-*Value] ^f,g^	Com Protect Rate [A vs. B, *p-*Value] ^f,g^
Study 1 [68]							
1A	Vaccinated	8	Half-match	Blood cells	FIV-Pet (20)	6/8 ^h^ ***[0.0070]***	6/8 ^g^ ***[0.0023]***
1B1	Vaccinated	2	Unrelated	Blood cells	FIV-Pet (20)	0/2 *[1.0000]*	0/9
1B2	Non-vaccinated	7	Half-match or Unrelated	Blood cells	FIV-Pet (20)	0/7	-
Study 2 [68]							
2A	Vaccinated	3	Half-match	Blood cells	FIV-Pet (50)	2/3 ^i^ *[0.0667]*	2/3 ^g^ *[0.3000]*
2B1	Vaccinated	2	Unrelated	Blood cells	FIV-Pet (50)	0/2	0/7
2B2	Non-vaccinated	5	Half-match or Unrelated	Blood cells	FIV-Pet (50)	0/5	-
Study 3 [68]							
3A	Vaccinated	2	Identical	Blood cells	FIV-Pet (100)	2/2	2/2 *[0.0667]*
3B	None	(4 Control cats)	NA	Blood cells	FIV-Pet (100)	0/4	0/4
Combined Partial-Complete Protection Vaccine Groups 1A + 2A + 3A: Combined Complete-Protection Vaccine Groups 1A + 2A + 3A: Combined Control Groups 1B + 2B + 3B:							10/13 ***[<0.0001]***
							6/13 ***[0.0013]*** 0/21
Study 4 [62]							
4A	Vaccinated	4	MLR-match	T cells	FIV-Pet (25)	3/4	3/4 *[0.1429]*
4B1	Vaccinated	2	MLR-match	B cells	FIV-Pet (25)	0/2	0/4
4B2	Non-vaccinated	2	Unrelated	PBMC	FIV-Pet (25)	0/2	-
Study 5 [62]							
5A1	Vaccinated	4	Complete-match	T cells	FIV-Pet (25)	4/4 ***[0.0286]***	8/10 ***[0.0419]***
5A2	Vaccinated	3	Partial or Complete-match	CD4^+^ T cells	FIV-Pet (25)	2/3 *[0.1429]*	-
5A3	Vaccinated	3	Partial or Complete-match	CD8^+^ T cells	FIV-Pet (25)	2/3 *[0.1429]*	-
5B1	Non-vaccinated	1	MLR-match	T cells	FIV-Pet (25)	0/1	0/4
5B2	Non-vaccinated	1	Unrelated	PBMC	FIV-Pet (25)	0/1	-
5B3	None	(2 Control cats)	NA	PBS	FIV-Pet (25)	0/2	-
Study 6 [62]							
6A1	Vaccinated	5	Complete-match	T cells	FIV-Pet (25)	5/5 ***[0.0079]***	6/7 *[0.0699]*
6A2	Vaccinated	2	Partial-match	T cells	FIV-Pet (25)	1/2 *[0.3333]*	-
6B1	Non-vaccinated	1	Unrelated	T cells	FIV-Pet (25)	0/1	0/4
6B2	None	(3 Control cats)	NA	PBS	FIV-Pet (25)	0/3	-
			Combined Complete Protection Vaccine Groups 5A1 + 6A1: Combined Control Groups 5B1 + 5B2 + 5B3 + 6B1 + 6B2:				9/9 *[0.0001]* 0/8
Study 7 [62]							
7A1	Vaccinated	9	Complete-match	T cells	FIV-FC1 (25)	6/9 ***[0.009]***	6/12 ***[0.0419]***
7A2	Vaccinated	3	Partial-match	T cells	FIV-FC1 (25)	0/3	-
7B1	Non-vaccinated	3	Unrelated	T cells	FIV-FC1 (25)	0/3	0/8
7B2	None	5	NA	PBS or None	FIV-FC1 (25)	0/5	-

^a^ Studies 1–3 and Studies 4–7 in Table 6 adapted and summarized from references [62,68], respectively. ^b^ Number of control cats (# Control cats) were without AT. ^c^ Half-match between parent to offspring; mixed leukocyte reaction (MLR) for MHC matching is a functional assay where more proliferation when MHC not matched; not applicable (NA). ^d^ PBS washed whole blood cells (Blood cells). ^e^ All studies with intravenous challenge at mean cat infectious dose (CID_50_). ^f^ Partial-Complete Protection (P-C Protect); Combined Protection (Com Protect); Studies A versus B [A vs. B]; two-tailed Fisher Exact Test for *p*-value (italics) with *p* < 0.05 as significant (bolded italics). ^g^ Only partial-complete protection combined for Studies 1 and 2; completed protection in Studies 3–6. ^h^ 3/8 complete protection and 3/8 partial protection; *p*-value comparing 1A vs. 1B2. ^i^ 1/3 complete protection and 1/3 partial protection; *p*-value comparing 2A vs. 2B2.

## References

[1] Pedersen N.C., Ho E.W., Brown M.L., Yamamoto J.K. (1987). Isolation of a T-Lymphotropic Virus from Domestic Cats with an Immunodeficiency-Like Syndrome. Science.

[2] Hoover E.A., Mullins J.I., Quackenbush S.L., Gasper P.W. (1987). Experimental Transmission and Pathogenesis of Immunodeficiency Syndrome in Cats. Blood.

[3] Yamamoto J.K., Sparger E., Ho E.W., Andersen P.R., O’Connor T.P., Mandell C.P., Lowenstine L., Munn R., Pedersen N.C. (1988). Pathogenesis of Experimentally Induced Feline Immunodeficiency Virus Infection in Cats. Am. J. Vet. Res..

[4] Bendinelli M., Pistello M., Lombardi S., Poli A., Garzelli C., Matteucci D., Ceccherini-Nelli L., Malvaldi G., Tozzini F. (1995). Feline Immunodeficiency Virus: An Interesting Model for AIDS Studies and an Important Cat Pathogen. Clin. Microbiol. Rev..

[5] Yamamoto J.K., Pedersen N.C., Ho E.W., Okuda T., Theilen G.H. (1988). Feline Immunodeficiency Syndrome—A Comparison between Feline T-Lymphotropic Lentivirus and Feline Leukemia Virus. Leukemia.

[6] Talbott R.L., Sparger E.E., Lovelace K.M., Fitch W.M., Pedersen N.C., Luciw P.A., Elder J.H. (1989). Nucleotide Sequence and Genomic Organization of Feline Immunodeficiency Virus. Proc. Natl. Acad. Sci. USA.

[7] Olmsted R.A., Hirsch V.M., Purcell R.H., Johnson P.R. (1989). Nucleotide Sequence Analysis of Feline Immunodeficiency Virus: Genome Organization and Relationship to Other Lentiviruses. Proc. Natl. Acad. Sci. USA.

[8] Olmsted R.A., Barnes A.K., Yamamoto J.K., Hirsch V.M., Purcell R.H., Johnson P.R. (1989). Molecular Cloning of Feline Immunodeficiency Virus. Proc. Natl. Acad. Sci. USA.

[9] Pecon-Slattery J., McCracken C.L., Troyer J.L., VandeWoude S., Roelke M., Sondgeroth K., Winterbach C., Winterbach H., O’Brien S.J. (2008). Genomic Organization, Sequence Divergence, and Recombination of Feline Immunodeficiency Virus from Lions in the Wild. BMC Genom..

[10] Ackley C.D., Yamamoto J.K., Levy N., Pedersen N.C., Cooper M.D. (1990). Immunologic Abnormalities in Pathogen-Free Cats Experimentally Infected with Feline Immunodeficiency Virus. J. Virol..

[11] Streeck H., Nixon D.F. (2010). T Cell Immunity in Acute HIV-1 Infection. J. Infect. Dis..

[12] Rosenberg Y.J., Janossy G. (1999). The Importance of Lymphocyte Trafficking in Regulating Blood Lymphocyte Levels during HIV and SIV Infections. Semin. Immunol..

[13] Sodora D.L., Allan J.S., Apetrei C., Brenchley J.M., Douek D.C., Else J.G., Estes J.D., Hahn B.H., Hirsch V.M., Kaur A. (2009). Toward an AIDS Vaccine: Lessons from Natural Simian Immunodeficiency Virus Infections of African Nonhuman Primate Hosts. Nat. Med..

[14] Hirsch V.M. (2004). What can Natural Infection of African Monkeys with Simian Immunodeficiency Virus Tell Us about the Pathogenesis of AIDS?. AIDS Rev..

[15] Daniel M.D., Letvin N.L., King N.W., Kannagi M., Sehgal P.K., Hunt R.D., Kanki P.J., Essex M., Desrosiers R.C. (1985). Isolation of T-Cell Tropic HTLV-III-Like Retrovirus from Macaques. Science.

[16] Gao F., Bailes E., Robertson D.L., Chen Y., Rodenburg C.M., Michael S.F., Cummins L.B., Arthur L.O., Peeters M., Shaw G.M. (1999). Origin of HIV-1 in the Chimpanzee Pan Troglodytes Troglodytes. Nature.

[17] Sharp P.M., Bailes E., Chaudhuri R.R., Rodenburg C.M., Santiago M.O., Hahn B.H. (2001). The Origins of Acquired Immune Deficiency Syndrome Viruses: Where and When?. Philos. Trans. R. Soc. Lond. B. Biol. Sci..

[18] Alter H.J., Eichberg J.W., Masur H., Saxinger W.C., Gallo R., Macher A.M., Lane H.C., Fauci A.S. (1984). Transmission of HTLV-III Infection from Human Plasma to Chimpanzees: An Animal Model for AIDS. Science.

[19] Pecon-Slattery J., Troyer J.L., Johnson W.E., O’Brien S.J. (2008). Evolution of Feline Immunodeficiency Virus in Felidae: Implications for Human Health and Wildlife Ecology. Vet. Immunol. Immunopathol..

[20] Troyer J.L., Pecon-Slattery J., Roelke M.E., Johnson W., VandeWoude S., Vazquez-Salat N., Brown M., Frank L., Woodroffe R., Winterbach C. (2005). Seroprevalence and Genomic Divergence of Circulating Strains of Feline Immunodeficiency Virus among Felidae and Hyaenidae Species. J. Virol..

[21] Yamamoto J.K., Pu R., Sato E., Hohdatsu T. (2007). Feline Immunodeficiency Virus Pathogenesis and Development of a Dual-Subtype Feline-Immunodeficiency-Virus Vaccine. AIDS.

[22] Roukaerts I.D.M., Theuns S., Taffin E.R.L., Daminet S., Nauwynck H.J. (2015). Phylogenetic Analysis of Feline Immunodeficiency Virus Strains from Naturally Infected Cats in Belgium and The Netherlands. Virus Res..

[23] Hohdatsu T., Fujimori S., Maeki M., Suma N., Motokawa K., Okada S., Koyama H. (1997). Virus Neutralizing Antibody Titer to Feline Immunodeficiency Virus Isolates of Subtypes A, B and D in Experimentally or Naturally Infected Cats. J. Vet. Med. Sci..

[24] Bachmann M.H., Mathiason-Dubard C., Learn G.H., Rodrigo A.G., Sodora D.L., Mazzetti P., Hoover E.A., Mullins J.I. (1997). Genetic Diversity of Feline Immunodeficiency Virus: Dual Infection, Recombination, and Distinct Evolutionary Rates among Envelope Sequence Clades. J. Virol..

[25] Hoover E.A., Mullins J.I., Chu H.-J., Wasmoen T.L. (1996). Efficacy of an Inactivated Feline Leukemia Virus Vaccine. AIDS Res. Hum. Retrovir..

[26] Pedersen N.C. (1993). Immunogenicity and Efficacy of a Commercial Feline Leukemia Virus Vaccine. J. Vet. Intern. Med..

[27] Patel M., Carritt K., Lane J., Jayappa H., Stahl M., Bourgeois M. (2015). Comparative Efficacy of Feline Leukemia Virus (FeLV) Inactivated Whole-Virus Vaccine and Canarypox Virus-Vectored Vaccine during Virulent FeLV Challenge and Immunosuppression. Clin. Vaccine Immunol..

[28] Torres A.N., O’Halloran K.P., Larson L.J., Schultz R.D., Hoover E.A. (2010). Feline Leukemia Virus Immunity Induced by Whole Inactivated Virus Vaccination. Vet. Immunol. Immunopathol..

[29] Kang C.Y., Gao Y. (2017). Killed Whole-HIV Vaccine; Employing a Well Established Strategy for Antiviral Vaccines. AIDS Res. Ther..

[30] Choi E., Michalski C.J., Choo S.H., Kim G.N., Banasikowska E., Lee S., Wu K., An H.Y., Mills A., Schneider S. (2016). First Phase I Human Clinical Trial of a Killed Whole-HIV-1 Vaccine: Demonstration of its Safety and Enhancement of Anti-HIV Antibody Responses. Retrovirology.

[31] International AIDS Vaccine Initiative, Database of Preventative HIV Vaccine Candidates. https://www.iavi.org/trials-database.

[32] Graziani G.M., Angel J.B. (2016). HIV-1 Immunogen: An Overview of Almost 30 Years of Clinical Testing of a Candidate Therapeutic Vaccine. Expert Opin. Biol. Ther..

[33] Yamamoto J.K. (2010). Evolving Perspectives on FIV Vaccines. Practitioner’s Updated: Feline Retrovirus Disease.

[34] Yamamoto J.K., Hansen H., Ho E.W., Morishita T.Y., Okuda T., Sawa T.R., Nakamura R.M., Pedersen N.C. (1989). Epidemiologic and Clinical Aspects of Feline Immunodeficiency Virus Infection in Cats from the Continental United States and Canada and Possible Mode of Transmission. J. Am. Vet. Med. Assoc..

[35] Goldkamp C.E., Levy J.K., Edinboro C.H., Lachtara J.L. (2008). Seroprevalences of Feline Leukemia Virus and Feline Immunodeficiency Virus in Cats with Abscesses or Bite Wounds and Rate of Veterinarian Compliance with Current Guidelines for Retrovirus Testing. J. Am. Vet. Med. Assoc..

[36] Vernazza P.L., Eron J.J., Fiscus S.A., Cohen M.S. (1999). Sexual Transmission of HIV: Infectiousness and Prevention. AIDS.

[37] Westman M.E., Paul A., Malik R., McDonagh P., Ward M.P., Hall E., Norris J.M. (2016). Seroprevalence of Feline Immunodeficiency Virus and Feline Leukaemia Virus in Australia: Risk Factors for Infection and Geographical Influences (2011–2013). JFMS Open Rep..

[38] Hosie M.J., Addie D., Belak S., Boucraut-Baralon C., Egberink H., Frymus T., Gruffydd-Jones T., Hartmann K., Lloret A., Marsilio F. (2009). Feline Immunodeficiency: ABCD Guidelines on Prevention and Management. J. Feline Med. Surg..

[39] O’Neil L.L., Burkhard M.J., Diehl L.J., Hoover E.A. (1995). Vertical Transmission of Feline Immunodeficiency Virus. AIDS Res. Hum. Retrovir..

[40] Wasmoen T., Armiger-Luhman S., Egan C., Hall V., Chu H.J., Chavez L., Acree W. (1992). Transmission of Feline Immunodeficiency Virus from Infected Queens to Kittens. Vet. Immunol. Immunopathol..

[41] Uhl E.W., Heaton-Jones T.G., Pu R., Yamamoto J.K. (2002). FIV Vaccine Development and its Importance to Veterinary and Human medicine: A Review FIV Vaccine 2002 Update and Review. Vet. Immunol. Immunopathol..

[42] Yamamoto J.K., Ackley C.D., Zochlinski H., Louie H., Pembroke E., Torten M., Hansen H., Munn R., Okuda T. (1991). Development of IL-2-independent Feline Lymphoid Cell Lines Chronically Infected with Feline Immunodeficiency Virus: Importance for Diagnostic Reagents and Vaccines. Intervirology.

[43] Huang C. (2002). Personal communication.

[44] Yamamoto J.K. (2002). Multi-Subtype FIV Vaccines. Application No. 09/512,746, filed Oct. 1, 1997. U.S. Patent.

[45] Yamamoto J.K., Hohdatsu T., Olmsted R.A., Pu R., Louie H., Zochlinski H.A., Acevedo V., Johnson H.M., Soulds G.A., Gardner M.B. (1993). Experimental Vaccine Protection Against Homologous and Heterologous Strains of Feline Immunodeficiency Virus. J. Virol..

[46] Yamamoto J.K., Okuda T., Ackley C.D., Louie H., Pembroke E., Zochlinski H., Munn R.J., Gardner M.B. (1991). Experimental Vaccine Protection Against Feline Immunodeficiency Virus. AIDS Res. Hum. Retrovir..

[47] Matteucci D., Poli A., Mazzetti P., Sozzi S., Bonci F., Isola P., Zaccaro L., Giannecchini S., Calandrella M., Pistello M. (2000). Immunogenicity of an Anti-Clade B Feline Immunodeficiency Fixed-Cell Virus Vaccine in Field Cats. J. Virol..

[48] Coleman J.K., Pu R., Martin M.M., Noon-Song E.N., Zwijnenberg R., Yamamoto J.K. (2014). Feline Immunodeficiency Virus (FIV) Vaccine Efficacy and FIV Neutralizing Antibodies. Vaccine.

[49] Uhl E.W., Martin M., Coleman J.K., Yamamoto J.K. (2008). Advances in FIV Vaccine Technology. Vet. Immunol. Immunopathol..

[50] Dunham S.P., Bruce J., MacKay S., Golder M., Jarrett O., Neil J.C. (2006). Limited Efficacy of an Inactivated Feline Immunodeficiency Virus Vaccine. Vet. Rec..

[51] Kusuhara H., Hohdatsu T., Okumura M., Sato K., Suzuki Y., Motokawa K., Gemma T., Watanabe R., Huang C., Arai S. (2005). Dual-Subtype Vaccine (Fel-O-Vax FIV) Protects Cats Against Contact Challenge with Heterologous Subtype B FIV Infected Cats. Vet. Microbiol..

[52] Huang C., Conlee D., Loop J., Champ D., Gill M., Chu H.J. (2004). Efficacy and Safety of a Feline Immunodeficiency Virus Vaccine. Anim. Health Res. Rev..

[53] Huang C., Conlee D., Gill M., Chu H.J. (2010). Dual-Subtype Feline Immunodeficiency Virus Vaccine Provides 12 Months of Protective Immunity against Heterologous Challenge. J. Feline Med. Surg..

[54] Westman M.E., Malik R., Hall E., Harris M., Norris J.M. (2016). The Protective Rate of the Feline Immunodeficiency Virus Vaccine: An Australian Field Study. Vaccine.

[55] Yamamoto J.K. (2000). Personal communication.

[56] Tellier M.C., Pu R., Pollock D., Vitsky A., Tartaglia J., Paoletti E., Yamamoto J.K. (1998). Efficacy Evaluation of Prime-Boost Protocol: Canarypoxvirus-Based Feline Immunodeficiency Virus (FIV) Vaccine and Inactivated FIV-Infected Cell Vaccine Against Heterologous FIV Challenge in Cats. AIDS.

[57] Pistello M., Cammarota G., Nicoletti E., Matteucci D., Curcio M., Del Mauro D., Bendinelli M. (1997). Analysis of the Genetic Diversity and Phylogenetic Relationship of Italian Isolates of Feline Immunodeficiency Virus Indicates a High Prevalence and Heterogeneity of Subtype B. J. Gen. Virol..

[58] Kakinuma S., Motokawa K., Hohdatsu T., Yamamoto J.K., Koyama H., Hashimoto H. (1995). Nucleotide Sequence of Feline Immunodeficiency Virus: Classification of Japanese Isolates into Two Subtypes which are Distinct from Non-Japanese Subtypes. J. Virol..

[59] Rerks-Ngarm S., Pitisuttithum P., Nitayaphan S., Kaewkungwal J., Chiu J., Paris R., Premsri N., Namwat C., de Souza M., Adams E. (2009). Vaccination with ALVAC and AIDSVAX to Prevent HIV-1 Infection in Thailand. N. Engl. J. Med..

[60] Hohdatsu T., Okada S., Motokawa K., Aizawa C., Yamamoto J.K., Koyama H. (1997). Effect of Dual-Subtype Vaccine Against Feline Immunodeficiency Virus Infection. Vet. Microbiol..

[61] Omori M., Pu R., Tanabe T., Hou W., Coleman J.K., Arai M., Yamamoto J.K. (2004). Cellular Immune Responses to Feline Immunodeficiency Virus (FIV) Induced by Dual-Subtype FIV Vaccine. Vaccine.

[62] Aranyos A.M., Roff S.R., Pu R., Owen J.L., Coleman J.K., Yamamoto J.K. (2016). An Initial Examination of the Potential Role of T-Cell Immunity in Protection against Feline Immunodeficiency Virus (FIV) Infection. Vaccine.

[63] Haynes B.F., Gilbert P.B., McElrath M.J., Zolla-Pazner S., Tomaras G.D., Alam S.M., Evans D.T., Montefiori D.C., Karnasuta C., Sutthent R. (2012). Immune-Correlates Analysis of an HIV-1 Vaccine Efficacy Trial. N. Engl. J. Med..

[64] De Souza M.S., Ratto-Kim S., Chuenarom W., Schuetz A., Chantakulkij S., Nuntapinit B., Valencia-Micolta A., Thelian D., Nitayaphan S., Pitisuttithum P. (2012). The Thai Phase III Trial (RV144) Vaccine Regimen Induces T Cell Responses that Preferentially Target Epitopes within the V2 Region of HIV-1 Envelope. J. Immunol..

[65] Forthal D.N., Gilbert P.B., Landucci G., Phan T. (2007). Recombinant gp120 Vaccine-Induced Antibodies Inhibit Clinical Strains of HIV-1 in the Presence of Fc Receptor-Bearing Effector Cells and Correlate Inversely with HIV Infection Rate. J. Immunol..

[66] Gilbert P.B., Peterson M.L., Follmann D., Hudgens M.G., Francis D.P., Gurwith M., Heyward W.L., Jobes D.V., Popovic V., Self S.G. (2005). Correlation Between Immunologic Responses to a Recombinant Glycoprotein 120 Vaccine and Incidence of HIV-1 Infection in a Phase 3 HIV-1 Preventive Vaccine Trial. J. Infect. Dis..

[67] Pitisuttithum P., Gilbert P., Gurwith M., Heyward W., Martin M., van Griensven F., Hu D., Tappero J.W., Choopanya K., Bangkok Vaccine Evaluation Group (2006). Randomized, Double-blind, Placebo-Controlled Efficacy Trial of a Bivalent Recombinant Glycoprotein 120 HIV-1 Vaccine among Injection Drug Users in Bangkok, Thailand. J. Infect. Dis..

[68] Pu R., Omori M., Okada S., Rine S.L., Lewis B.A., Lipton E., Yamamoto J.K. (1999). MHC-Restricted Protection of Cats Against FIV Infection by Adoptive Transfer of Immune Cells from FIV-Vaccinated Donors. Cell. Immunol..

[69] Roff S.R., Noon-Song E.N., Yamamoto J.K. (2014). The Significance of Interferon-γ in HIV-1 Pathogenesis, Therapy, and Prophylaxis. Front. Immunol..

[70] Levy J.A. (1998). HIV and the Pathogenesis of AIDS.

[71] Huang Y., Duerr A., Frahm N., Zhang L., Moodie Z., De Rosa S., McElrath M.J., Gilbert P.B. (2014). Immune-Correlates Analysis of an HIV-1 Vaccine Efficacy Trial Reveals an Association of Nonspecific Interferon-γ Secretion with Increased HIV-1 Infection Risk: A Cohort-Based Modeling Study. PLoS ONE.

[72] Tanabe T., Yamamoto J.K. (2001). Feline Immunodeficiency Virus Lacks Sensitivity to the Antiviral Activity of Feline IFN-Gamma. J. Interferon Cytokine Res..

[73] Yamamoto J.K., Barré-Sinoussi F., Bolton V., Pedersen NC., Gardner M.B. (1986). Human Alpha- and Beta-Interferon but not Gamma- Suppress the In Vitro Replication of LAV, HTLV-III, and ARV-2. J. Interferon Res..

[74] Yamamoto J.K. (2013). Personal communication.

[75] Abbas A.K., Lichtman A.H., Pillai S. (2015). Cellular and Molecular Immunology.

[76] Ferguson A.L., Mann J.K., Omarjee S., Ndung’u T., Walker B.D., Chakraborty A.K. (2013). Translating HIV Sequences into Quantitative Fitness Landscapes Predicts Viral Vulnerabilities for Rational Immunogen Design. Immunity.

[77] Leslie A.J., Pfafferott K.J., Chetty P., Draenert R., Addo M.M., Feeney M., Tang Y., Holmes E.C., Allen T., Prado J.G. (2004). HIV Evolution: CTL Escape Mutation and Reversion after Transmission. Nat. Med..

[78] Borthwick N., Ahmed T., Ondondo B., Hayes P., Rose A., Ebrahimsa U., Hayton E.J., Black A., Bridgeman A., Rosario M. (2014). Vaccine-Elicited Human T Cells Recognizing Conserved Protein Regions Inhibit HIV-1. Mol. Ther..

[79] Abbott J.R., Sanou M.P., Coleman J.K., Yamamoto J.K. (2011). Evolutionarily Conserved T-Cell Epitopes on FIV for Designing an HIV/AIDS Vaccine. Vet. Immunol. Immunopathol..

[80] Sanou M.P., De Groot A.S., Murphey-Corb M., Levy J.A., Yamamoto J.K. (2012). HIV-1 Vaccine Trials: Evolving Concepts and Designs. Open AIDS J..

[81] Kunwar P., Hawkins N., Dinges W.L., Liu Y., Gabriel E.E., Swan D.A., Stevens C.E., Maenza J., Collier A.C., Mullins J.I. (2013). Superior Control of HIV-1 Replication by CD8+ T Cells Targeting Conserved Epitopes: Implications for HIV Vaccine Design. PLoS ONE.

[82] Sáez-Cirión A., Sinet M., Shin S.Y., Urrutia A., Versmisse P., Lacabaratz C., Boufassa F., Avettand-Fènoël V., Rouzioux C., Delfraissy J.F. (2009). Heterogeneity in HIV Suppression by CD8 T Cells from HIV Controllers: Association with Gag-Specific CD8 T Cell Responses. J. Immunol..

[83] Edwards B.H., Bansal A., Sabbaj S., Bakari J., Mulligan M.J., Goepfert P.A. (2002). Magnitude of Functional CD8+ T-Cell Responses to the Gag Protein of Human Immunodeficiency Virus Type 1 Correlates Inversely with Viral Load in Plasma. J. Virol..

[84] Klein M.R., van Baalen C.A., Holwerda A.M., Kerkhof Garde S.R., Bende R.J., Keet I.P., Eeftinck-Schattenkerk J.K., Osterhaus A.D., Schuitemaker H., Miedema F. (1995). Kinetics of Gag-Specific Cytotoxic T Lymphocyte Responses during the Clinical Course of HIV-1 Infection: A Longitudinal Analysis of Rapid Progressors and Long-Term Asymptomatics. J. Exp. Med..

[85] Sahay B., Nguyen C.Q., Yamamoto J.K. (2017). Conserved HIV Epitopes for an Effective HIV Vaccine. J. Clin. Cell. Immunol..

[86] Elyar J.S., Tellier M.C., Soos J.M., Yamamoto J.K. (1997). Perspectives on FIV Vaccine Development. Vaccine.

[87] Roff S.R., Sanou M.P., Rathore M.H., Levy J.A., Yamamoto J.K. (2015). Conserved Epitopes on HIV-1, FIV and SIV p24 Proteins are Recognized by HIV-1 Infected Subjects. Hum. Vaccines Immunother..

[88] Sanou M.P., Roff S.R., Mennella A., Sleasman J.W., Rathore M.H., Yamamoto J.K., Levy J.A. (2013). Evolutionarily Conserved Epitopes on Human Immunodeficiency Virus Type 1 (HIV-1) and Feline Immunodeficiency Virus Reverse Transcriptases Detected by HIV-1-Infected Subjects. J. Virol..

[89] Sahay B., Yamamoto J.K. Lessons Learned from Developing a Commercial FIV Vaccine. Proceedings of the International Conference on Vaccines and Immunology.

